# Ectoparasite- and Vector-Borne-Related Dermatoses: A Single-Centre Study with Practical Diagnostic and Management Insights in a One Health Perspective

**DOI:** 10.3390/jcm15020851

**Published:** 2026-01-20

**Authors:** Giovanni Paolino, Barbara Moroni, Antonio Podo Brunetti, Anna Cerullo, Carlo Mattozzi, Giovanni Gaiera, Manuela Cirami, Dino Zilio, Mario Valenti, Andrea Carugno, Giuseppe Esposito, Nicola Zerbinati, Carmen Cantisani, Franco Rongioletti, Santo Raffaele Mercuri, Matteo Riccardo Di Nicola

**Affiliations:** 1 Unit of Dermatology and Cosmetology, IRCCS San Raffaele Hospital, Via Olgettina 60, 20132 Milan, Italy; paolino.giovanni@hsr.it (G.P.); mercuri.santoraffaele@hsr.it (S.R.M.); 2 Istituto Zooprofilattico Sperimentale del Piemonte, Liguria e Valle d’Aosta, Via Bologna 148, 10154 Turin, Italy; barbara.moroni@izsplv.it (B.M.); anna.cerullo@izsplv.it (A.C.); giuseppe.esposito@izsplv.it (G.E.); 3 Dermatology Clinic, IRCCS San Raffaele Hospital, 20132 Milan, Italy; a.podobrunetti@studenti.unisr.it (A.P.B.); rongioletti.franco@hsr.it (F.R.); 4 Private Medical Practice, Via Vittorio Veneto 148, 91026 Mazara, Italy; carlo.mattozzi@gmail.com; 5 Clinic of Infectious Diseases, IRCCS San Raffaele Scientific Institute, 20132 Milan, Italy; gaiera.giovanni@hsr.it; 6 Plastic and Reconstructive Surgery Unit, IRCCS Policlinico San Donato, Piazza Edmondo Malan 2, 20097 San Donato Milanese, Italy; manuelacirami@gmail.com (M.C.); dzilio.plastic@libero.it (D.Z.); 7 Dermatology Unit, IRCCS Humanitas Research Hospital, 20089 Rozzano, Italy; mario.valenti@hunimed.eu; 8 Department of Medicine and Innovation Technology (DiMIT), University of Insubria, 21100 Varese, Italy; andrea.carugno@uninsubria.it (A.C.); nicola.zerbinati@uninsubria.it (N.Z.); 9 Department of Dermatology, Sapienza University of Rome, Viale del Policlinico 155, 00161 Rome, Italy; carmencantisanister@gmail.com; 10 Faculty of Medicine, Vita-Salute San Raffaele University, 20132 Milan, Italy

**Keywords:** Acari, antiparasitic resistance, arthropod-borne dermatoses, dermoscopy, Dirofilaria, Insecta, Nematoda, non-invasive diagnostics, parasitic skin diseases, reflectance confocal microscopy

## Abstract

**Background**: Parasitic skin-related conditions represent a frequent and evolving challenge in human dermatology, as they often mimic other dermatoses, and are increasingly complicated by therapeutic resistance. With this study, we aimed to provide a practical, clinician-oriented overview of our experience, contextualising it within the current literature. **Materials and Methods**: We conducted a retrospective, single-centre observational study, reporting a case series of 88 patients diagnosed with parasitic or arthropod-related skin infestations at the San Raffaele Hospital Dermatology Unit (Milan) between 2019 and 2024, and integrated a concise narrative review of contemporary evidence on diagnosis, non-invasive imaging and management. For each case, we documented clinical presentation, dermoscopic or reflectance confocal microscopy (RCM) findings, and treatment response. Non-invasive tools (dermoscopy, videodermoscopy, RCM) were used when appropriate. **Results**: The spectrum of conditions included flea bites, bed bug bites, cutaneous larva migrans, subcutaneous dirofilariasis, *Dermanyssus gallinae* dermatitis, pediculosis, tick bites (including Lyme disease), myiasis, scabies, and cutaneous leishmaniasis. One case of eosinophilic dermatosis of haematologic malignancy was also considered due to its possible association with arthropod bites. Non-invasive imaging was critical in confirming suspected infestations, particularly in ambiguous cases or when invasive testing was not feasible. Several cases highlighted suspected therapeutic resistance: a paediatric pediculosis and three adult scabies cases required systemic therapy after standard regimens failed, raising concerns over putative resistance to permethrin and pyrethroids. In dirofilariasis, the persistence of filarial elements visualised by RCM justified the extension of antiparasitic therapy despite prior surgical removal. **Conclusions**: Our findings underline that accurate diagnosis, early intervention, and tailored treatment remain essential for the effective management of cutaneous infestations. The observed vast spectrum of isolated parasites reflects broader health and ecological dynamics, including zoonotic transmission, international mobility, and changing environmental conditions. At the same time, diagnostic delays, inappropriate treatments, and neglected parasitic diseases continue to pose significant risks. To address these challenges, clinicians should remain alert to atypical presentations, and consider a multidisciplinary approach including the consultation with parasitologists and veterinarians, as well as the incorporation of high-resolution imaging and alternative therapeutic strategies into their routine practice.

## 1. Introduction

Cutaneous infections and infestations are widespread, although some conditions show geographical predominance. Climate change, immigration, international trade and travel, as well as the increasing ownership of pet animals, may all contribute to a higher risk of contracting these infestations [1]. For this reason, in the modern era, it is no longer appropriate to consider certain infestations as confined to specific geographic regions. Instead, these conditions should be approached from a more global perspective, as clinicians are increasingly encountering diagnoses that would have been considered rare or unlikely in their particular geographic areas in the past [2,3,4,5].

While in some cases the diagnosis is straightforward due to the presence of characteristic lesions (e.g., linear and migratory lesions in larva migrans), or the presence of the parasite itself, in other cases, the lesions can mimic other dermatoses. In these instances, confirmation through specific laboratory investigations, including microbiological, histopathological, serological, and radiological tests, is warranted. However, laboratory investigations have limitations, such as delayed results, the need for specialised infrastructures and trained personnel, and patient reluctance to undergo invasive procedures like skin biopsy. These factors often present practical challenges for clinicians, especially in resource-limited healthcare settings [6]. Consequently, the use of non-invasive diagnostic methods (such as dermoscopy, videodermoscopy and reflectance confocal microscopy—RCM) may assist both patients and clinicians in reaching a timely diagnosis.

Parasitic and arthropod-related cutaneous infestations can manifest with a spectrum of dermatological symptoms, including pruritus, erythema, papular eruptions, ulcerations, and secondary bacterial infections [3,4,5,7]. Early diagnosis is critical for effective management, as misdiagnosis or delayed treatment can lead to complications and prolonged suffering for affected individuals, as well as the increased risk of transmission to other people. Furthermore, the increasing prevalence of treatment-resistant infestations, such as scabies unresponsive to standard therapies, underscores the need for continued research and novel therapeutic strategies [4,8].

Given the above, timely and accurate diagnosis remains central to clinical management, as it enables the prompt initiation of targeted therapy, reduces transmissibility, and minimises the risk of recurrence. Accordingly, the integration of well-trained dermatological expertise with non-invasive diagnostic tools helps refine case recognition and improve patient outcomes. This work focuses on three emerging and clinically relevant aspects: (i) the growing challenge of therapeutic resistance, (ii) the evolving role of advanced non-invasive methods in routine dermatology practice, and (iii) the increasing emergence of previously neglected zoonotic diseases (such as scabies and leishmaniasis) in humans. In this regard, it is critical to ensure a strong interface between dermatologists, general practitioners, veterinarians, and parasitologists, in order to enable the early diagnosis of these skin conditions through a multidisciplinary approach, in the “One Health” perspective. By presenting a single-centre case series, integrated by a concise narrative review of the literature, we illustrate how these issues manifest in real-world patients with parasitic and arthropod-related dermatoses. The cases, commonly encountered in outpatient settings, depict everyday clinical scenarios and also highlight suspected resistance or treatment-failure patterns that may compromise conventional approaches, as well as situations in which dermoscopy, videodermoscopy, or reflectance confocal microscopy can be critical for diagnosis. Through comprehensive evaluation, diagnostic workup, and therapeutic management, we aim to expand current understanding of these conditions, highlighting the current improved diagnostic methodologies and more effective, targeted therapeutic strategies.

## 2. Materials and Methods

This retrospective, single-centre study, complemented by a concise narrative review, included consecutive patients with parasitic and arthropod-related dermatoses seen at the Dermatology Unit of San Raffaele Hospital (Milan, Italy) between January 2019 and December 2024.

Specifically, we included patients with a diagnosis of a parasitic or arthropod-related dermatosis, namely cutaneous infestations caused by ectoparasites (e.g., scabies, pediculosis, phthiriasis), cutaneous forms of vector-borne infections (e.g., leishmaniasis, tick-borne diseases, cutaneous dirofilariasis), and eosinophilic dermatosis of haematological malignancy (EDHM). We chose to retain EDHM within the spectrum of arthropod-related dermatoses because this entity, historically described as an insect bite-like reaction or exaggerated arthropod-bite lesion in patients with haematologic malignancies, presents with intensely pruritic papulo-vesicular eruptions on exposed sites, shows a clear seasonal pattern, and exhibits clinical and histopathologic features that closely mimic arthropod bites, with growing clinico-pathological evidence supporting a triggering role of insect bites in its pathogenesis [9,10,11]. In order to be included in the study, all patients were required to have a complete or sufficiently detailed electronic medical record, including clinical history, dermatologic examination, diagnosis of parasitic-related dermatosis, and treatment information. Exclusion criteria included patients with an unconfirmed or uncertain diagnosis, in which the suspicion of a parasitic or arthropod-related dermatosis could not be supported by adequate clinical, dermoscopic, or laboratory evidence. Cases with incomplete medical documentation that did not allow retrieval of key variables such as diagnostic details, treatment information, or clinical outcomes were excluded from the analyses, as well as cases of systemic parasitic infections without any cutaneous involvement, as the study focused exclusively on dermatologic manifestations of parasitic and arthropod-related conditions.

Electronic medical records were reviewed to extract age, sex, lesion type and characteristics, topical and/or systemic treatments, concomitant therapies/comorbidities, country of likely acquisition, and country of diagnosis/treatment; when available, clinical photographs, dermoscopic findings and RCM findings were also reviewed. Non-invasive imaging included handheld polarised dermoscopy (Heine Delta 20; Heine Optotechnik GmbH & Co. KG, Gilching, Germany), videodermoscopy (Vidix 4.0; Deka M.E.L.A. s.r.l., Calenzano, Italy), and in vivo horizontal RCM (VivaScope 1500 and VivaScope 3000; VivaScope GmbH, Munich, Germany). Ectoparasites, when present on the patient, were isolated and morphologically identified first under the stereomicroscope (Zeiss Discovery V12, Jena, Germany). Then, when further morphological details were necessary, they were observed under the light microscope (Axio Zeiss Imager A1, Jena, Germany) after placing them in lactophenol at 45 ◦C for a week. For the morphological identification, published taxonomical keys were used for each ectoparasite [12,13,14].

In cases where further clinical procedures or laboratory analyses were necessary to establish a final diagnosis (e.g., Lyme disease, leishmaniasis), their outcomes were recorded.

Treatment efficacy and outcomes were assessed by condition and therapeutic approach. Suspected treatment resistance was defined as persistence or recurrence of active infestation after guideline-concordant first-line therapy with documented adherence, requiring escalation to systemic and/or combination therapy. Treatment failure was defined as the persistence or recurrence of clinically and/or dermoscopically confirmed active infestation following completion of guideline-concordant first-line therapy, in the absence of identifiable alternative explanations.

An example of stepwise diagnostic workflow applied in the present work is summarised in Figure S1.

The study was a retrospective review in which all variables were irreversibly anonymised at source before extraction and analysis, with no re-identification key retained and no procedures beyond standard care. Data extracted from electronic medical records were independently reviewed and cross-checked by three authors (GP, CM and MRDN) with specific expertise in parasitic and arthropod-related dermatoses to ensure accuracy and internal consistency. Any discrepancies were resolved by consensus, and, when necessary, by re-evaluating the original clinical documentation together with available non-invasive imaging data (dermoscopy, videodermoscopy, and RCM). All data were irreversibly anonymised prior to analysis, and completeness and plausibility checks were performed before inclusion in the final dataset. All arthropod drawings are original digital illustrations created by the authors; they were produced de novo using multiple photographic references and were not traced from any single source. All photographs used in the collages were taken by the authors with the patients’ informed consent. Given the retrospective design, treatment adherence was assessed indirectly using a composite approach based on routine clinical documentation. Adherence was inferred from: documentation in the electronic medical record of treatment completion as reported by the patient at follow-up; absence of reported deviations from prescribed dosage, duration, or application method; review of prescription records when available and exclusion of common confounding factors such as incorrect application, premature discontinuation, or failure to treat close contacts and household members when indicated. Cases in which adherence could not be reasonably assessed, or where reinfestation or improper treatment execution was suspected, were not classified as treatment failures. The term “resistance” was used descriptively to indicate clinical non-response requiring escalation of therapy, without implying microbiologically or molecularly confirmed drug resistance.

To contextualise the case series, we conducted a brief, targeted narrative review of contemporary evidence on diagnosis, non-invasive imaging and management of dermatoses caused by ectoparasites or vector-borne diseases with cutaneous forms. We performed non-systematic searches of PubMed/MEDLINE and Scopus, and screened key dermatology and infectious-disease guidelines up to August 2025. Search strategies combined condition terms (for example, scabies/Sarcoptes scabiei; pediculosis capitis/corporis; phthiriasis pubis; bed bug bites/*Cimex lectularius/C. hemipterus*; flea bites/Pulex; cutaneous larva migrans/Ancylostoma; subcutaneous dirofilariasis/*Dirofilaria repens/D. immitis*; *Dermanyssus gallinae* dermatitis; tick bites and Lyme borreliosis; cutaneous myiasis; cutaneous leishmaniasis/Leishmania; eosinophilic dermatosis of haematological malignancy) with diagnostic and management-related terms (dermoscopy, videodermoscopy, reflectance confocal microscopy/RCM, entodermoscopy, diagnosis, management, treatment/therapy, guidelines, resistance, pyrethroid resistance, permethrin failure, ivermectin, albendazole, malathion). As examples, the following search strings were used in PubMed/MEDLINE: for scabies: (“scabies” OR “*Sarcoptes scabiei*”) AND (“dermoscopy” OR “reflectance confocal microscopy” OR “diagnosis” OR “treatment” OR “permethrin” OR “ivermectin” OR “resistance” OR “treatment failure”); for pediculosis: (“pediculosis” OR “*Pediculus humanus capitis*” OR “head lice”) AND (“dermoscopy” OR “diagnosis” OR “treatment” OR “pyrethroid resistance” OR “ivermectin” OR “malathion”).

The literature review was clinician-oriented and was not intended to be systematic or comprehensive; searches were performed to support clinical contextualisation and discussion rather than to generate pooled epidemiological estimates or formal evidence synthesis. Articles were selected based on clinical relevance, recency, and applicability to dermatological practice, with priority given to recent reviews, guidelines, and clinically oriented studies. No formal systematic screening or quality assessment was performed, in line with the narrative nature of the review.

The study was conducted in accordance with the principles of the Declaration of Helsinki and with institutional policies on the use of retrospective clinical data for research purposes. As this was a retrospective observational study based exclusively on anonymised data derived from routine clinical practice, without any additional or invasive procedures, formal approval by an ethics committee was not required under current local regulations.

## 3. Results

Parasitic and arthropod-related dermatoses analysed in this study included flea bites, bed bug bites, cutaneous larva migrans (CLM), subcutaneous dirofilariasis, *Dermanyssus gallinae* dermatitis, pediculosis, tick bites (including Lyme disease), myiasis, scabies (all with suspected drug-resistance), and cutaneous leishmaniasis; EDHM was also briefly discussed. All included cases derived from routine outpatient practice, thus reflecting common clinical scenarios. A total of 88 patients were included (mean age 28.8 years; 54.5% females) (Table 1 and Table S1). The most frequent conditions were pediculosis (36.4%), tick bites/Lyme disease (28.4%), and bed bug bites (20.5%), followed by flea bites (4.5%), scabies (3.4%), and other parasitic dermatoses such as cutaneous larva migrans, subcutaneous dirofilariasis, *Dermanyssus gallinae* dermatitis, myiasis, cutaneous leishmaniasis, and EDHM (each 1.1%). Overall, pediculosis was the most prevalent infestation, predominantly affecting children (mean age 9 years) and females (87.5%). Tick bites occurred mainly in adults (mean age 46 years), with Lyme disease confirmed in 28% of tick-related cases. Suspected therapeutic resistance was documented in 4/88 patients (4.5%), specifically one case of pediculosis (3.1% of lice infestations) and three cases of scabies (100% of scabies cases).

To aid rapid clinical consultation, Table 2 summarises the recent literature on epidemiology, clinical and diagnostic features, reported suspected resistance or treatment failure, and standard therapies for the entomodermatoses documented in the present series.

Below, we report individual cases of infestation according to the specific parasitic disease and, when available, based on the identified pathogen.

### 3.1. Flea Bites

Flea infestations are prevalent worldwide, particularly in warm, humid environments. They often affect households with domestic animals infested with fleas and are a well-known cause of pruritic eruptions, especially in children [15].

**Figure 1:**
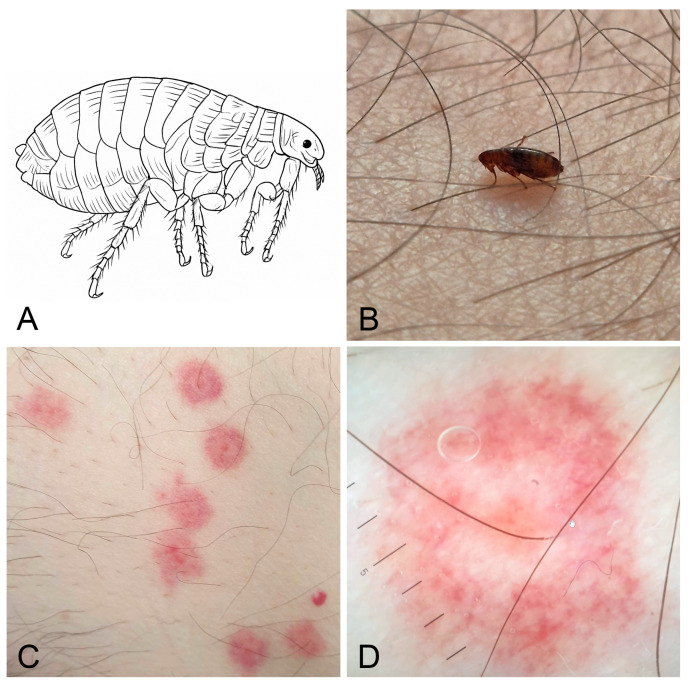
Flea bites. (**A**) Simplified lateral view drawing of *Pulex irritans* (order Siphonaptera). (**B**) *Pulex irritans* feeding on a leg. (**C**) Clinical presentation on the leg: multiple central puncta with surrounding ecchymotic and purpuric haloes caused by digestive enzymes. (**D**) Dermoscopic view of flea bites. Image quality is limited by the resolution of the original clinical documentation; the image is retained for its educational value.

Flea bites are a relatively frequent dermatological condition, especially in rural areas or in settings characterised by overcrowding, caused by fleas (Siphonaptera: Pulicidae), which are obligatory hematophagous insects (Figure 1A,B).

Clinically, the bites present as grouped, erythematous, pruritic papules, often with a central punctum. They are typically located on the lower limbs or other areas exposed to infested animals. A characteristic central puncture surrounded by an ecchymotic and purpuric halo, due to the action of digestive enzymes, is often observed (Figure 1C). Dermoscopy reveals annular macules with an ecchymotic appearance and central microerosion corresponding to the bite site (Figure 1D) [16]. In sensitised individuals, lesions may evolve into papular urticaria or flea allergy dermatitis (FAD), with possible secondary bacterial infection resulting from scratching [17].

Treatment is primarily symptomatic and includes topical corticosteroids and oral antihistamines to manage inflammation and pruritus. In cases of superinfection, topical or systemic antibiotics may be required. Effective management also requires the identification and treatment of infested animals, along with environmental pest control [17,18].

In our department we observed four cases (three males and one female; mean age: 32 years), all infested with *Pulex irritans* fleas, the human flea, although often reported in other animal species such as wildlife and livestock [69,70]. The patients reported having had contact with pet animals or living in the same household with them. All cases were successfully treated with corticosteroids, antihistamines, repellents, and environmental pest control. No cases of suspected treatment resistance were recorded. The patients did not report any additional symptoms such as fever or unusual pain; therefore, no further diagnostic investigations were performed.

### 3.2. Bed Bug Bites

Bed bug infestations have increased globally since the 1990s, particularly in urban areas and high-density housing. They are not limited by hygiene and affect hotels, dormitories, and hospitals. Their resurgence has been linked to insecticide resistance and increased international travel [20].

**Figure 2:**
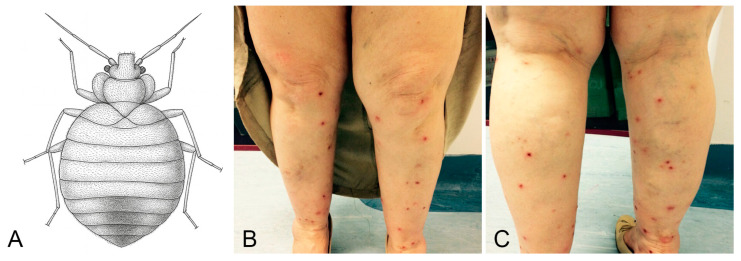
Bed bug bites. (**A**) Simplified dorsal view drawing of *Cimex lectularius* (order *Hemiptera*). (**B**) Linearly distributed excoriated papules on the anterior aspect of the legs. (**C**) Same lesions on the posterior aspect of the legs. Image quality is limited by the resolution of the original clinical documentation; the image is retained for its educational value.

Bed bugs (*Cimex lectularius* and *Cimex hemipterus*; Hemiptera: Cimicidae) are nocturnal hematophagous insects (Figure 2A) that infest human dwellings and feed on exposed skin, causing pruritic, erythematous papules often arranged in linear patterns (commonly described as the “breakfast, lunch, and dinner” sign) (Figure 2B,C).

Diagnosis is clinical and relies on patient history, typical skin lesions, and identification of environmental infestation. Although the skin reactions are self-limiting, symptomatic management with topical corticosteroids, oral antihistamines or gabapentinoids is often required [21].

Eradication requires professional pest control using an integrated strategy that includes laundering, vacuuming, application of extreme temperatures, and insecticides. Increasing resistance to commonly used agents, such as pyrethroids highlights the importance of tailored, multi-modal extermination plans [22].

In our clinic, we treated 18 patients (8 males and 10 females; mean age: 32 years), all with a combination of topical therapy, systemic antihistamines, and environmental pest control. All patients reported intense pruritus during or after the night, and specimens of bed bugs were recovered in some cases.

### 3.3. Cutaneous Larva Migrans

Cutaneous larva migrans (CLM) is most frequently encountered in tropical and subtropical regions worldwide, particularly in Africa, South America, the Far East, and the South Pacific; in Europe, cases are predominantly reported in travellers returning from endemic areas such as Malaysia, Thailand, Indonesia, Brazil, the Caribbean, and West Africa [23].

**Figure 3:**
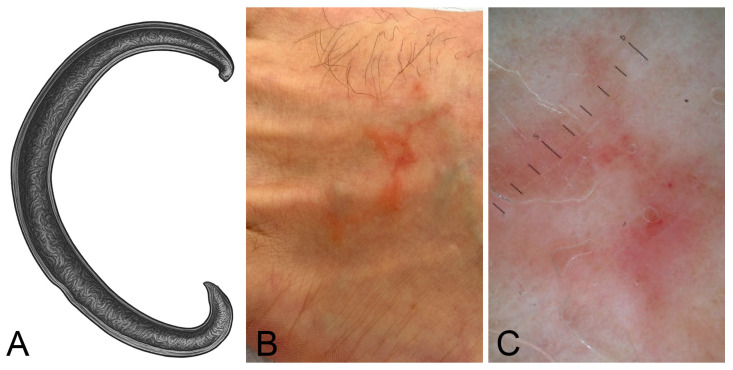
Larva migrans. (**A**) Simplified drawing of *Ancylostoma* sp. (**B**) Serpiginous, erythematous tracks on the skin, accompanied by intense pruritus and localised inflammation. (**C**) Dermoscopy showed erythematous and serpiginous tracks. Image quality is limited by the resolution of the original clinical documentation; the image is retained for its educational value.

CLM, in human patients, is a parasitic skin infection caused by hookworm larvae (Figure 3A), most commonly *Ancylostoma braziliense* (Nematoda: Ancylostomatidae). Accurate species identification would require genetic analysis of the larval material. However, given the clinical urgency and the fact that treatment does not vary significantly among causative species, genetic typing is typically omitted in routine care.

Clinically, CLM presents with serpiginous, erythematous tracks on the skin, accompanied by intense pruritus and localised inflammation (Figure 3B,C). These tracks result from the subcutaneous migration of larvae, which are unable to invade deeper tissues in humans [24]. Diagnosis is primarily clinical, based on lesion morphology and travel or exposure history.

First-line treatment includes oral albendazole (400 mg daily for 3–5 days) or ivermectin; mebendazole is considered an alternative [25]. Environmental management and pest control also play a role in preventing recurrence.

We treated a 52-year-old man who presented with clinical manifestation 30 days after returning from a holiday in Brazil. The patient was initially treated with topical corti-costeroids at another hospital, without improvement. At our first consultation, he reported pruritus and exhibited bilateral, whitish, serpiginous cutaneous lesions. Dermoscopy confirmed the presence of a whitish, scaly, serpiginous track. A clinical diagnosis of larva migrans was made, and treatment with albendazole 400 mg daily for 7 days was performed. At a 2-month follow-up, the patient had completely recovered and was symptom-free.

### 3.4. Subcutaneous Dirofilariasis

Human dirofilariasis is a zoonotic infection caused by nematodes of the genus *Dirofilaria* (Nematoda: Onchocercidae), transmitted through mosquito bites. The two main species involved are *Dirofilaria repens*, typically causing subcutaneous or ocular nodules, and, more rarely, *Dirofilaria immitis*, which may lead to pulmonary lesions [26,27,71].

Clinically, in human patiens, *D. repens* infections often present as single, painless subcutaneous nodules, frequently located on the face or extremities. Rarely, atypical sites such as the eye, lungs, or central nervous system may be involved. Humans do not usually develop microfilaremia, as the parasite is unable to mature into adult worms and reach sexual maturity. Diagnosis can be confirmed via histopathological examination of excised tissue. Ideally, precise identification would require genetic testing of the collected parasite, although unfortunately this is often omitted due to the urgency of clinical intervention and because treatment does not vary substantially between species. Surgical excision remains the treatment of choice; antiparasitic therapy is not routinely indicated, though it may be considered in selected cases [28,29,30].

**Figure 4:**
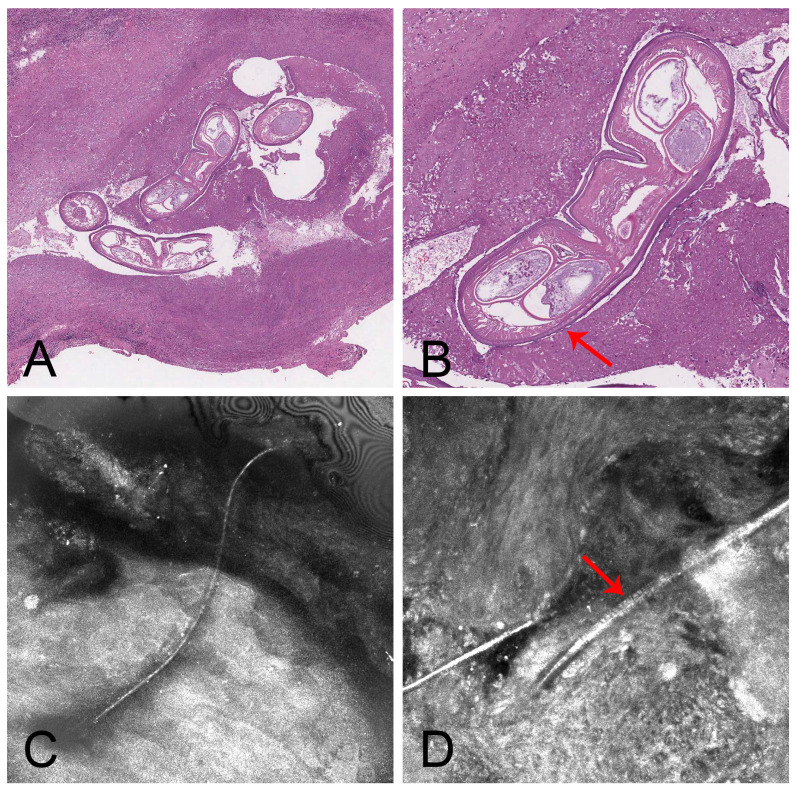
*Dirofilaria* sp. (**A**) Histology showed the presence of a nematode (Hematoxylin and Eosin, 100×). (**B**) At higher magnification, the nematode structure are evident: thick cuticle, muscle layer, body cavity and oviducts. The helminth is surrounded by an intense inflammatory infiltrate, mainly characterised by plasma cells and eosinophils (Hematoxylin and Eosin, 200×). The red arrow indicates the external longitudinal ridge. (**C**) RCM basic image of upper layers of the epidermis shows the presence of multiple filiform elements. (**D**) RCM basic image of lower layers of the epidermis and superficial dermis shows well-circumscribed, cylindrical-shaped elements, characterised by external multiple wavy ridges and central body cavity. Numerous hypereflective cells are also present and compatible with the infiltrate of plasma cells and eosinophils. The red arrow indicates the external longitudinal ridge.

We report the case of a 72-year-old woman with no significant medical history who presented to our Dermatology Unit for the evaluation of a painless subcutaneous neoformation on the right temporal region, which had developed over the preceding five months, in absence of other evident cutaneous changes. The patient was afebrile and without any other symptoms. A cutaneous ultrasound was performed, revealing only a nonspecific and hypoechoic subcutaneous lesion. Consequently, a surgical excision of the lesion was performed. Histological examination revealed, in the superficial dermis, a mixed granulomatous and chronic inflammatory reaction with the presence of plasma cells and eosinophils surrounding a morphologically compatible nematode parasite (Figure 4A,B). Therefore, further exploration of the patient’s clinical history was conducted. The patient, residing in Calabria (Southern Italy), reported no recent travel to tropical locations but did mention a brief camping in Portugal three months earlier. Based on the anatomical location of the subcutaneous lesion in the delicate right temporal region, we decided to avoid a wide excision to conduct further investigations (e.g., molecular detection from unfixed material and assessment of possible helminth persistence in the surrounding tissue). Considering the potential persistence of the helminths after the surgical excision and involvement of the surrounding area, an inspection was performed using the in vivo RCM. RCM of the skin adjacent to the excised area revealed the presence of multiple filiform elements characterised by external multiple “wavy” ridges and central body cavity (Figure 4C,D; https://youtu.be/5dcnvI-riVw accessed on 30 September 2025) consistent in general morphology and size with a juvenile stage, of an Onchocercidae Nematoda [31,32]. The morphological identification of the filarial larval stage could not be achieved at the species level. Nevertheless, based on the localization of the nodule, the species was presumed to be *D. repens*.

A treatment with albendazole 800 mg daily for 28 days was prescribed. After a follow-up of 2 months, RCM showed the absence of helminths and ophthalmological examination ruled out ocular dirofilariasis.

### 3.5. Dermanyssus gallinae Dermatitis

*Dermanyssus gallinae* (Acari: Dermanyssidae), commonly known as the red poultry mite, is an avian ectoparasite that primarily infests birds. Typical reservoirs include poultry houses and pens, bird nests located on air conditioning units, window ledges, and building façades [36]. When deprived of their natural hosts, these mites may migrate into nearby buildings and infest mammals, including domestic pets [35] and humans [72].

**Figure 5:**
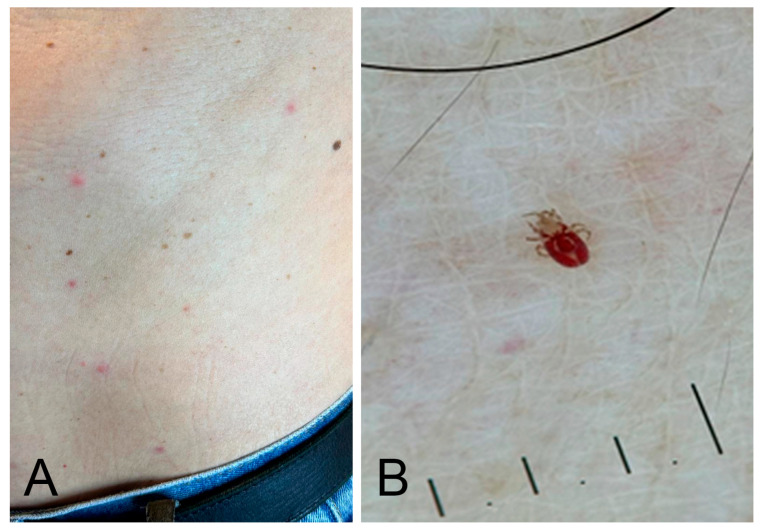
*Dermanyssus gallinae*. (**A**) Clinical: maculo-papular lesions of the trunk. (**B**) *D. gallinae* dermoscopy. Image quality is limited by the resolution of the original clinical documentation; the image is retained for its educational value.

Clinically, infestations cause pruritic macular, papular, or vesicular lesions, often localised to covered areas like the trunk and nuchal region (Figure 5A,B) [36].

These manifestations are frequently misdiagnosed as scabies, trombiculosis, or other zoonotic mite-related dermatoses [55]. Diagnosis is confirmed by microscopic identification of the mite from skin or environmental samples. Treatment involves eliminating the source of infestation, thorough environmental cleaning, and disinfection of clothing and bedding using hot water and acaricides [37,38]. Patients are advised to take baths rather than showers to fully remove mites. Symptomatic relief can be achieved with antihistamines and topical corticosteroids. Although the mite does not reproduce on humans, some authors recommend topical permethrin (1–5%) in persistent or severe cases. Cohabitants should receive the same environmental and symptomatic care if heavily exposed [39].

We observed a case in a 70-year-old male patient residing in the Sicilian countryside, who presented with intensely pruritic lesions on the trunk and limbs (Figure 5). Complete resolution of the cutaneous manifestations was achieved following topical treatment, environmental pest control, and management of farm and companion animals.

### 3.6. Pediculosis

**Figure 6:**
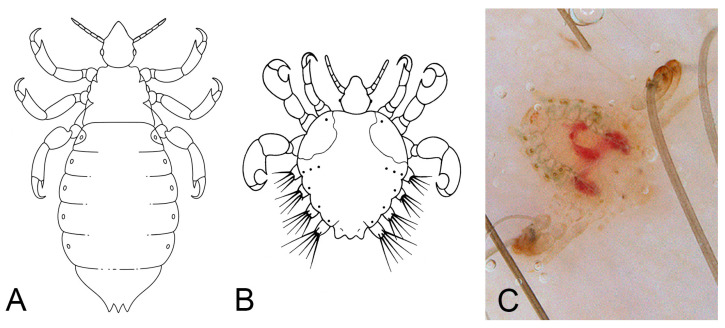
Lice. Simplified dorsal view drawings of (**A**) *Pediculus humanus capitis* and (**B**) *Pthirus pubis*. (**C**) Dermoscopy of *Pthirus pubis* attached to pubic hair. Image quality is limited by the resolution of the original clinical documentation; the image is retained for its educational value.

Pediculosis refers to the infestation of humans by obligate ectoparasites such as lice, most commonly *Pediculus humanus capitis* (head lice) and *Pediculus humanus corporis* (body lice; Phthiraptera: Pediculidae), and *Pthirus pubis* (crab lice; Phthiraptera: Pthiridae) (Figure 6A–C) [40].

Head lice are most common in children and typically cause scalp pruritus, excoriations, and cervical lymphadenopathy. Body lice are known vectors of serious diseases such as epidemic typhus and trench fever. Pubic lice are usually sexually transmitted and may be indicative of other sexually transmitted infections [41]. Treatment involves mechanical removal (e.g., wet combing) and the use of chemical pediculicides such as permethrin, malathion, pyrethrins, or lindane. Resistance to these agents is an increasing concern and may require alternating or combining therapies. Environmental control, such as laundering clothing and bed linens and vacuuming, is essential, especially in cases of body lice infestation. Pubic lice are typically treated with 5% permethrin cream; infestation of the eyelashes can be managed with occlusive agents such as petroleum jelly [42].

In our clinic, we recorded 32 cases of pediculosis (mean age: 9 years; 28 females and 4 males). Among the paediatric pediculosis cases, one patient exhibited persistent infestation despite repeated first-line treatment. The child had a history of recurrent head lice over several months, with documented exposure in a school setting and simultaneous infestation of close contacts. Prior treatments included multiple applications of topical permethrin, reportedly administered according to instructions, with only temporary improvement. At presentation, live lice and viable nits were still observed, supporting a diagnosis of treatment failure rather than reinfestation alone. A combined regimen of topical permethrin and oral ivermectin was therefore initiated, resulting in complete eradication of infestation at follow-up.

### 3.7. Tick Bites and Lyme Disease

**Figure 7:**
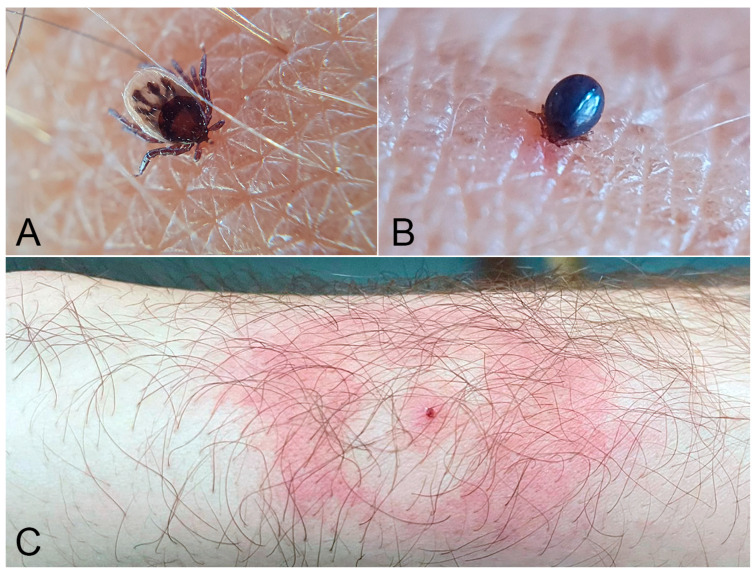
*Ixodes ricinus*. (**A**) Nymph before engorgement. (**B**) Engorged nymph after blood meal. (**C**) Cutaneous annular erythema at the tick bite site. Image quality is limited by the resolution of the original clinical documentation; the image is retained for its educational value.

Lyme disease, caused by *Borrelia burgdorferi* sensu lato complex and transmitted by *Ixodes* ticks (Acari: Ixodidae) (Figure 7A,B), is endemic in temperate regions of North America, Europe, and Asia.

The disease progresses through distinct clinical stages, beginning with localised erythema migrans (Figure 7C), followed by disseminated involvement with neurologic and cardiac manifestations, and potentially culminating in late-stage arthritis. Diagnosis is largely clinical, especially in the presence of the hallmark skin lesion, and is confirmed using a two-tiered serological approach [47].

Treatment depends on the stage of the disease. Doxycycline is the first-line therapy for early localised and mild neurologic or cardiac involvement, while intravenous ceftriaxone is preferred for severe neurologic involvement or high-degree heart block. Most patients respond well to appropriate antibiotic therapy; however, a subset may develop antibiotic-refractory arthritis due to immune-mediated mechanisms rather than persistent infection [48].

In our clinic, we observed a total of 25 tick-bite cases (18 males, 7 females; mean age 46 years). In 16 patients, the tick was still attached at presentation, whereas 4 patients presented with a target-like erythema at the bite site and 5 reported having removed the tick themselves before the visit (none of the latter subsequently tested positive for *Borrelia*). Overall, 7 patients (5 males, 2 females; mean age 48 years) had serologically confirmed Borrelia burgdorferi infection, and an erythema migrans-like lesion was documented in 5 of these 7 cases. One of these patients, a 46-year-old male, developed atrioventricular block and myocarditis, which resolved following treatment. These complications occurred despite appropriate doxycycline therapy; in retrospect, the patient reported taking most doses together with dairy products, a practice that may have reduced antibiotic absorption and contributed to a suboptimal early response [73,74]. Ticks retrieved from the patients, either collected directly as specimens or provided indirectly by the patients, were morphologically identified as *Ixodes ricinus* (one of the most common tick species in Europe, also known as the castor bean tick). All specimens were nymphs or adult females. In several cases, the patients’ history included recurrent hiking in mountainous and wooded areas with their pets, which is consistent with the likely source of infestation.

### 3.8. Cutaneous Myiasis

The infestation of live vertebrate animals with dipterous larvae, which for at least a certain period feed on the host’s dead or living tissues, body fluids, or ingested food, is defined as myiasis. Dipteran flies causing myiasis are classified as obligate or facultative parasites; in both cases, the larvae feed on necrotic tissues or body fluids of the host for a variable period. Once mature, the larvae leave the host to pupate in the environment, where they develop into adult flies (imagines).

**Figure 8:**
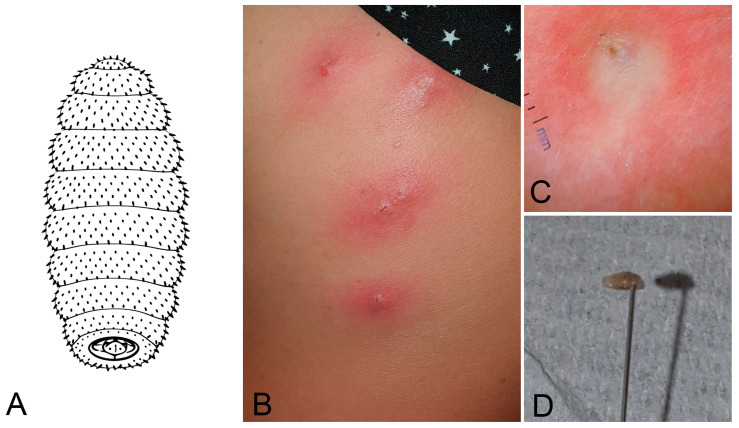
Cutaneous myiasis. (**A**) Simplified drawings of a *Cordylobia anthropophaga* larva. (**B**) Erythematous nodular lesions with central pore of the dorsum. (**C**) Dermoscopy of the larval pore surrounded by the erythematous lesions. (**D**) Larva extracted from the patient. Image quality is limited by the resolution of the original clinical documentation; the image is retained for its educational value.

Human cutaneous myiasis is more common in tropical and rural areas and typically affects individuals exposed to flies during travel or outdoor activities. *Dermatobia hominis* (Diptera: Oestridae) in Central/South America and *Cordylobia anthropophaga* (Diptera: Calliphoridae) in Africa are among the main causative species of human obligate cutaneous myiasis [51], although other genera and species such as *Lucilia sericata* (Diptera: Calliphoridae), *Calliphora* spp. (Diptera: Calliphoridae), *Sarcophaga* spp. (Diptera: Sarcophagidae; Figure 8A) and *Wohlfahrtia magnifica* (Diptera: Sarcophagidae) are also reported as facultative zoonotic myiasis. In Europe, most cases are imported in travellers; autochthonous cutaneous/wound myiasis is mainly linked to calliphorids and *W. magnifica*, with rare reports due to *Hypoderma lineatum* (Diptera: Oestridae).

This parasitic skin condition is caused by dipteran larvae infesting living tissue and typically presents as furuncular, migratory, or wound-associated lesions. Clinically, it manifests as erythematous nodules with a central pore, which may discharge serous fluid and be associated with pruritus, localised pain, or a sensation of movement (Figure 8B,C) [52].

Diagnosis is primarily clinical but can be supported by dermoscopy or ultrasonography in unclear cases. Treatment focuses on larval removal through occlusion techniques (e.g., petroleum jelly or occlusive dressings), which induce larval emergence by obstructing respiration. Manual extraction is commonly performed using forceps or pressure after local anaesthesia, with care taken to avoid larval rupture. In selected cases, surgical intervention may be required. Ivermectin, administered either topically or orally, serves as an effective larvicidal agent, particularly in extensive or anatomically difficult infestations. Prompt treatment minimises the risk of secondary bacterial infection, granuloma formation, and scarring, with most lesions healing completely after larval removal [53,54].

We report the case of a 41-year-old Caucasian woman who presented with multiple furuncular lesions characterised by central openings (ostia). Dermoscopy revealed the presence of live, motile larvae. The nodules were incised with a surgical blade, and a larva was extracted using needles. The wounds were subsequently disinfected with povidone–iodine, and prophylactic antibiotic therapy was prescribed. The patient had recently returned from a trip to Congo (Central Africa), where *Cordylobia anthropophaga* is endemic and represents a frequent cause of furuncular myiasis in residents and travellers [75,76].

### 3.9. Scabies

**Figure 9:**
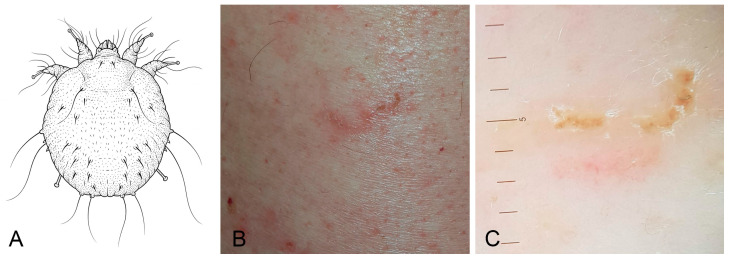
Scabies. (**A**) Simplified dorsal view drawing of *Sarcoptes scabiei*. (**B**) Clinical presentation: excoriated papules and burrows on the back. (**C**) Dermoscopic view of a scabietic patient. Image quality is limited by the resolution of the original clinical documentation; the image is retained for its educational value.

Scabies is a skin infestation caused by the mite *Sarcoptes scabiei* var *hominis* (Sarcoptiformes: Sarcoptidae) which burrows into the stratum corneum of the epidermis (Figure 9A–C). Scabies affects over 130 million people globally, with highest prevalence in tropical regions and resource-limited settings. Transmission occurs primarily through direct skin-to-skin contact, although fomite transmission is also possible under specific environmental conditions. The infestation typically causes intense pruritus and inflammatory lesions, which may be complicated by secondary bacterial infections [4,56,57,58]. Episodes of zoonotic transmission are increasingly reported, although usually animal-derived scabies in humans is described as a self-limiting condition [55].

Therapeutic failure in scabies is increasingly recognised as a major contributor to its global resurgence. A recent systematic review estimated an overall treatment failure rate of 15.2%, with higher rates observed for older agents such as benzyl benzoate (25.3%) and crotamiton (27.7%), while permethrin and oral ivermectin had lower but rising failure rates over time. Notably, two doses of oral ivermectin significantly reduced failure rates compared to a single dose (7.1% vs. 15.2%) [59]. Despite growing concern, drug resistance remains poorly characterised, partly due to the lack of standardized in vivo susceptibility testing in clinical trials, and because patients often fail to apply topical treatments correctly. The rising rates of treatment failure, particularly for permethrin, along with regional and temporal variability, suggest a potential decline in mite susceptibility. Treatment optimisation, including repeated dosing and combination regimens, should be considered in resistant cases [60].

In our clinic, we observed three cases of drug-resistant scabies, all in male patients (mean age: 24 years). These patients presented with persistent pruritus and typical scabietic lesions despite prior treatment. All patients reported close-contact exposure with symptomatic people, and in two cases intrafamilial transmission was documented. Before referral to our centre, patients had received one or more courses of topical benzyl benzoate (10–25%), with incomplete or transient clinical response. At presentation, active infestation was confirmed both, clinically and dermoscopically, with persistence of burrows and pruritus, suggesting true treatment failure rather than reinfestation or improper application. Given the persistence of active disease, treatment was escalated to combined topical permethrin and oral ivermectin, leading to complete clinical resolution. The condition was successfully resolved using a combination of topical permethrin and oral ivermectin. However, these observations should be interpreted as descriptive clinical findings rather than evidence of a broader resistance trend, given the absence of a control group and inferential analysis.

### 3.10. Cutaneous Leishmaniasis

**Figure 10:**
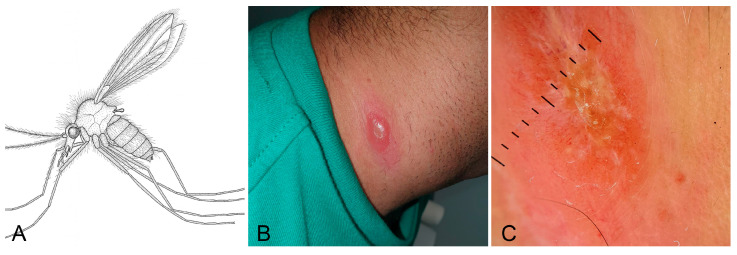
*Leishmaniasis*. (**A**) Simplified lateral view drawing of a female phlebotomine sandfly, the primary vector of *Leishmania* spp. (**B**) Clinical presentation of a cutaneous leishmaniasis lesion at the base of the neck: well-demarcated erythematous papule with central ulceration and raised borders. (**C**) Dermoscopic image showing a central yellowish area with a white starburst pattern, surrounded by dotted and linear vessels and peripheral hyperkeratosis. Image quality is limited by the resolution of the original clinical documentation; the image is retained for its educational value.

Cutaneous leishmaniasis (CL) is a parasitic skin disease caused by protozoa of the genus *Leishmania* (Kinetoplastida: Trypanosomatidae), transmitted to humans through the bite of infected female sandflies of the genera *Phlebotomus* (Old World) or *Lutzomyia* (New World) (Diptera: Psychodidae; Figure 10A) [65,66,67]. Clinically, it manifests as one or multiple ulcerative skin lesions, often located on exposed areas such as the face or limbs, with variable morphology depending on the Leishmania species involved and host immune response.

Lesions typically begin as papules and progress to painless ulcers with raised borders; in some cases, atypical presentations such as verrucous, eczematous or nodular forms may occur. The disease is endemic in various regions of the Mediterranean basin, the Middle East, South America, and parts of Asia and Africa. In Europe, *L. infantum* is the most commonly implicated species, often with zoonotic transmission involving dogs as reservoirs [61,62,63,64,65].

Diagnosis is clinical, supported by patient history (e.g., travel to endemic areas or exposure to infected vectors) and confirmed by parasitological methods (direct microscopy, PCR, or culture from lesion samples). Dermoscopy can aid diagnosis by revealing central ulceration, peripheral hyperkeratosis, and vascular structures, although findings are not pathognomonic [63].

Treatment depends on lesion severity, number, location, and species involved. Therapeutic options include systemic or topical antimonials, amphotericin B, miltefosine, or cryotherapy for selected cases. Healing may occur spontaneously but can leave disfiguring scars [68]. A 30-year-old male patient presented with a papulo-ulcerative lesion on the neck, present for approximately one month. A skin biopsy was performed, and histopathological examination was consistent with cutaneous leishmaniasis. Dermoscopic evaluation revealed a central yellowish area with a “yellow tears” pattern and an ulcerated component (Figure 10B,C). The patient was treated with one vial of medication administered weekly for four consecutive weeks, achieving complete resolution of the lesion within 30 days.

### 3.11. Eosinophilic Dermatosis of Haematologic Malignancy: The Role of Insect Bites

EDHM is an uncommon skin condition primarily observed in patients with haematologic cancers, particularly chronic lymphocytic leukaemia (CLL) [11,77]. Although historically considered an exaggerated hypersensitivity reaction to insect bites, the role of insects has remained controversial due to inconsistent patient recollection of exposure [77,78]. However, recent evidence supports a potential etiologic link: in a retrospective study of 35 EDHM patients, 94% showed seasonal lesion onset, and lesions frequently occurred on exposed areas such as limbs, aligning with typical insect bite distribution [10]. Histopathologic features, including dense eosinophilic infiltrates and flame figures, closely resemble those seen in arthropod bite reactions. Additionally, overlooked arthropods such as mites may contribute to underdiagnosis of bite-induced dermatoses [11,77,79,80,81].

These findings underscore a potential triggering role of insect bites in EDHM within a context of immune dysregulation, supporting the hypothesis that EDHM may represent a spectrum disorder related to arthropod hypersensitivity and adaptive immune imbalance [11]. In our sample we observed a 51-year-old male patient with CLL who died 20 days after the onset of EDHM.

Unlike the other conditions included in this series, EDHM does not represent a true parasitic or vector-borne disease, as no infectious agent is involved. Its inclusion in the present study reflects its well-documented clinicopathological overlap with arthropod bite reactions and its frequent clinical misinterpretation as a parasitic infestation. In this context, EDHM exemplifies a distinct diagnostic subgroup, in which insect bites are considered a potential triggering factor acting on a background of profound immune dysregulation, rather than a direct causative agent. This distinction is clinically relevant, as misclassification may lead to inappropriate antiparasitic treatments and diagnostic delays.

## 4. Discussion

Cutaneous infestations remain a recurring challenge in dermatological practice. This is particularly evident in the context of shifting epidemiological dynamics, growing global mobility, and the emergence of resistant parasites. In this series, drawn from routine cases in a tertiary dermatology centre, we present a varied sample of infestations across diverse demographic, environmental settings, managed within a multidisciplinary framework. The observed conditions ranged from frequently encountered disorders, such as pediculosis and scabies, to less common parasitic diseases like cutaneous larva migrans, dirofilariasis, and myiasis.

When compared with available regional and European epidemiological data, the distribution of cases observed in our series appears broadly consistent with previously reported patterns. To contextualize our findings, we compared our case distribution with recent Italian and European epidemiological data. In Italy and across Europe, several recent studies and public health reports have documented a renewed increase in scabies after the COVID-19 period, with hospital- and community-based series showing a marked rise in diagnoses [82,83]. Pediculosis capitis remains highly prevalent in school-aged children, with Italian public health surveys and European prevalence studies consistently reporting recurrent outbreaks and substantial under-ascertainment in routine surveillance [84]. Regarding Lyme borreliosis, EU/EEA surveillance data and Italian notification systems confirm marked geographical heterogeneity and under-reporting, supporting the broad consistency of our observations with current regional trends [46].

Similarly, the relatively lower number of scabies cases reflects the outpatient, non-institutional setting of our cohort, as higher scabies prevalence is typically reported in long-term care facilities, shelters, and resource-limited environments [8]. Imported or travel-related conditions such as cutaneous larva migrans, myiasis, and cutaneous leishmaniasis were infrequent but expected findings in a tertiary referral centre, mirroring their low incidence but increasing recognition in Southern Europe [65,85]. Overall, while the present series cannot be considered epidemiologically representative, its case spectrum aligns with broader regional trends, supporting the external plausibility of the observed distribution.

Non-invasive diagnostic tools, particularly dermoscopy and RCM, played a central role in confirming infestation and guiding management in this series, especially in clinically ambiguous cases. In addition, for more ambiguous presentations, particularly those involving deeper structures, RCM adds a layer of diagnostic confidence. In our suspected case of dirofilariasis, RCM enabled the visualisation of residual filiform elements morphologically compatible with larval stages of filarial nematodes. This confirmed the parasitic origin and helped guide further management. These observations underscore the clinical value of integrating high-resolution imaging techniques into routine assessment, especially in cases where standard examination is inconclusive or limited by lesion location. Nonetheless, while accurate diagnosis is a prerequisite for effective management, our series also highlights that correct identification does not invariably translate into therapeutic success.

Indeed, although ectoparasitic infestations can be accurately diagnosed through morphological identification, their management is not always straightforward, as cases of relapse or resistance to pharmacological treatment may occur, requiring escalation beyond first-line therapy. Specifically, three young adult males in our cohort failed to respond to benzyl benzoate and required escalation to oral ivermectin and topical permethrin. These findings are consistent with recent reports pointing to a gradual but measurable reduction in the effectiveness of widely used scabicides. A systematic review estimated a treatment failure rate of over 15%, with evidence of rising resistance, particularly to permethrin, over the last decade. Although resistance remains difficult to quantify due to the lack of standardised in vivo testing methods, the trend is concerning and warrants closer surveillance [59,86]. Similarly, in our paediatric pediculosis cases, one child exhibited resistance requiring systemic therapy. This aligns with the increasing number of reports describing widespread pyrethroid resistance in *Pediculus humanus capitis*. A 2023 meta-analysis estimated global resistance to pyrethroids at nearly 60%, with rates as high as 82% in post-2015 studies. This is likely related to sodium channel mutations (T929I, L932F) that impair the neurotoxic action of the compounds [43,44]. Interestingly, studies based on genetic markers report higher resistance rates than those based on clinical outcomes, suggesting underrecognition in everyday settings. Alternative approaches, such as physically acting agents (dimethicone, isopropyl myristate) and non-pyrethroid treatments like ivermectin or malathion, are increasingly relevant, especially in recurrent cases. Emerging products, including essential oils and silicon-based formulations, have shown promise in preliminary studies, though widespread adoption awaits further validation. These considerations reinforce the need to reconsider treatment protocols, especially in light of environmental and behavioural factors that may also reduce therapeutic success. Bed bug infestations, which accounted for 18 cases in our cohort, continue to pose practical management and environmental challenges. Although symptomatic relief can often be achieved with standard treatments, eradication remains difficult, particularly in settings where pyrethroid resistance is prevalent. Environmental control plays a central role, but the growing inefficacy of conventional insecticides has led to a shift toward integrated pest management strategies [45]. Patients should also be advised to avoid bringing untreated second-hand furniture into the home and to reduce clutter, which creates ideal harbourage sites. Although simple in theory, these measures are often overlooked or inconsistently applied, which may explain the recurrence seen in some of our patients.

The issue of resistance extends beyond treatment failures and into the field of prevention. Recent studies have reported that some pyrethroid-resistant mosquito and bed bug populations are less sensitive to commonly used repellents, including DEET and IR3535. These findings raise the possibility that chemical resistance mechanisms may cross-affect both insecticides and repellents. In response, researchers are exploring spatial repellents, which function by altering insect behaviour at a distance, and bio-derived compounds such as monoterpenes and sesquiterpenes that target different molecular pathways. While still in development, these alternative strategies may help compensate for the declining effectiveness of conventional repellents [87,88,89].

At least eight of the conditions observed in our cohort, namely cutaneous larva migrans, dirofilariasis, leishmaniasis, myiasis, flea bites, *Dermanyssus*-related dermatosis, pseudoscabies and tick bites represent zoonotic infections. In these cases, although genetic identification of the causative species would have offered greater taxonomic precision, and additional information on the putative source of infestation, it was not necessary for patient management. Treatment was guided by clinical presentation and parasite morphological identification, with albendazole and ivermectin proving sufficient in most cases [90].

In the case of human dirofilariasis, however, additional antiparasitic therapy was administered after histological and RCM findings revealed residual parasitic elements in surrounding tissues, despite excision of the visible lesion. This case illustrates the potential for ongoing parasitic activity even after apparent surgical cure and supports the use of imaging tools in therapeutic follow-up.

Our series also included one case of *D. gallinae* infestation, a diagnosis frequently missed due to its clinical overlap with scabies and pseudoscabies. The patient, who lived in a rural area, had been exposed to birds. Symptoms resolved following removal of the source and targeted treatment. The case underscores the importance of considering mite infestations in the differential diagnosis of persistent pruritic dermatoses, especially in patients from environments with bird nests or poultry.

Lyme disease was another relevant condition observed, with seven patients testing positive for *Borrelia burgdorferi* and one developing cardiac complications. Notably, that patient had taken doxycycline with dairy products, a well-documented interaction that can impair drug absorption. The case highlights the need for clear communication with patients regarding drug interactions, particularly when managing diseases like Lyme borreliosis, where treatment timing and bioavailability are critical. Secondary bacterial infection remains a common concern in parasitic infestations, especially when pruritus, excoriation, or breach of the skin barrier is involved. In selected cases from our cohort, including myiasis and extensive flea bites, prophylactic antibiotics were administered to reduce the risk of superinfection. Although no universal guidelines exist for prophylaxis in this context, clinicians should consider it in immunocompromised individuals or when lesions are extensive or ulcerated. The decision to use topical or systemic agents should be based on the patient’s overall condition, lesion burden, and infection risk [91,92].

Cutaneous leishmaniasis (CL) is a neglected tropical disease caused by various Leishmania species, transmitted to humans through the bite of infected female phlebotomine sandflies. The primary reservoir of *Leishmania* spp. is the dog, although the role of various wildlife species remains a matter of debate [93]. The disease is endemic in more than 90 countries across the Americas, Africa, the Middle East, Central Asia, and the Mediterranean basin. The World Health Organization estimates 600,000–1 million new cases annually, although underreporting is common. In recent years, the global incidence of CL has shown an upward trend, partly due to climate change, increased human mobility, urbanization, and expansion of vector habitats into previously non-endemic areas. A number of environmental and anthropogenic factors are progressively reshaping the patterns of exposure to vector species. Among these, the movement of potentially infected animals across regions, the gradual expansion of urban areas into previously rural and endemic zones, and the broader effects of climate change significantly alter the distribution, abundance, and seasonal activity of competent vectors. These dynamics contribute to the emergence or re-emergence of leishmaniasis in areas where it was once sporadic, and they facilitate the persistence of transmission cycles in regions already affected. Understanding how these drivers interact is crucial for predicting shifts in disease epidemiology and for developing effective surveillance and control strategies [61].

Clinically, CL manifests as chronic papules, nodules, or ulcers that may heal spontaneously but often result in disfiguring scars. While the disease is rarely life-threatening, its psychosocial impact can be substantial.

Pentavalent antimonials, such as sodium stibogluconate and meglumine antimoniate, remain first-line therapy in many regions. However, treatment failures and drug resistance have been increasingly reported, especially in parts of the Indian subcontinent and the Middle East. Resistance mechanisms include decreased drug uptake, enhanced efflux, and thiol-mediated detoxification. Alternative therapies (such as liposomal amphotericin B, miltefosine) and local treatments (e.g., cryotherapy, thermo-therapies) are essential options, particularly in resistant or relapsing cases. Strengthening surveillance and access to effective treatments remains critical in curbing the rising global burden of CL.

Finally, we documented one case of EDHM in a patient with CLL. To avoid misclassification, EDHM was intentionally analysed outside the main parasitic case series, as it represents a reactive dermatosis mimicking arthropod infestations rather than a true infectious or zoonotic condition. Indeed, while the exact role of arthropod bites in EDHM remains under debate, the seasonal pattern and lesion distribution observed in this and other cases support the possibility of a hypersensitivity reaction triggered by unnoticed insect exposure. The interplay between immune dysregulation and external environmental factors may therefore play a more central role than previously appreciated. The main differential diagnoses of EDHM include arthropod bite reactions, scabies (and pseudoscabies), papular urticaria, bullous pemphigoid (pre-bullous phase), and drug-induced hypersensitivity reactions.

In the patients’ anamnestic history, the presence of animals, whether pets, livestock, or wildlife, here reported in several of the described cases, highlights a potential source of infestation. This information is crucial for prompting a rapid diagnosis and for guiding appropriate management of the infestation source, which may be environmental or animal in origin (e.g., fleas, ticks, scabies mites, *Dermanyssus* spp.).

Moreover, it is well known that many zoonotic ectoparasitoses and arthropod-borne-associated diseases are far more prevalent in animal hosts than in humans (e.g., pulicosis, leishmaniosis). As a consequence, human cases often represent incidental or secondary events arising from an undiagnosed or untreated animal reservoir. This underscores the crucial role of veterinary medicine in supporting human healthcare, particularly in ensuring timely diagnosis, identification of the source of infestation, and appropriate elimination or treatment of the animal host. Strengthening collaboration between veterinary and human medical professionals is therefore essential to interrupt transmission chains, prevent reinfestation, and achieve effective and sustained disease control. This principle underscores the One Health perspective, which recognises the interconnectedness of human, animal, and environmental health. Integrating information on patients’ exposure to animals and their habitats enables a more effective clinical assessment, supports the early detection of zoonotic or vector-borne risks, and facilitates targeted preventive measures.

Our observations underscore the importance of integrating precise diagnostic assessment with flexible, case-adapted therapeutic strategies, while acknowledging the descriptive nature of the present study. In any case, given the retrospective and descriptive design of this study, all observations should be interpreted as hypothesis-generating and context-dependent, without implying causal relationships or population-level trends.

A limitation of this study is its retrospective design and the indirect assessment of treatment adherence, which relied on patient recall and the completeness of clinical documentation. Consequently, cases classified as treatment failure or suspected resistance should be interpreted as pure clinical-based observation, with limiting factors related to possible self or environmental reinfestation, or incorrect application of topical therapy. Therefore, these findings should not be considered as evidence of true pharmacological resistance, but rather as real-world clinical findings in which actual treatment exposure cannot be fully verified. In addition, detailed anamnestic and exposure data were not available across all cases, including information on household contacts, animal exposure, environmental conditions, and potential sources of infestation. This incompleteness limits the reconstruction of source of infestation, the assessment of reinfestation risk, and the interpretation of outcomes within a broader epidemiological context; accordingly, conclusions should be interpreted with caution and generalisability remains limited.

## 5. Conclusions

The cases analysed in this retrospective study, considered alongside a brief narrative review anchored to a single-centre case series, reflect the evolving complexity of cutaneous infestations, which increasingly intersect with issues of diagnostic accuracy, suspected drug resistance, and changing environmental exposures. While many of the conditions encountered remain clinically familiar, their presentation and management are no longer straightforward. The growing role of non-invasive imaging tools, together with the need for updated therapeutic protocols and resistance surveillance, highlights how even common infestations require a more nuanced approach. A careful clinical history, awareness of overlooked etiologies, and attention to emerging resistance patterns are essential components of effective management. By synthesising contemporary evidence with local experience, this brief review also underscores the importance of integrating entomodermatoses into broader public-health discussions, particularly in light of changing ecological dynamics and the declining efficacy of conventional control measures, and points to priorities for surveillance and standardised definitions of treatment failure.

To translate the One Health perspective into routine clinical practice, simple and feasible operational tools should be integrated into dermatology outpatient workflows. We propose the use of a structured exposure checklist during the dermatologic consultation, systematically recording contact with pets or livestock, bird nests or poultry, rural environments, travel history, and household infestations. In suspected zoonotic or environmentally mediated dermatoses, this checklist could activate a standardised referral pathway with a veterinarian or local animal health service to identify and manage the potential source of infestation. In parallel, patients should be informed that recurrent or treatment-refractory infestations often reflect an untreated animal or environmental reservoir and require simultaneous management of both the patient and the exposure source.

## Figures and Tables

**Table 1:** Ectoparasite and vector-borne-related dermatoses managed at the Dermatology Unit, San Raffaele Hospital (Milan), 2019–2024. M = Male; F = Female.

Infestation Type	No. of Patients	Mean Age (Years)	Gender (M/F)	Treatment(s)	Suspected Resistance
Flea bites	4	32	3 M/1 F	Repellents, topical corticosteroids, antihistamines, pest control	No
Bed bug bites	18	32	8 M/10 F	Repellents, topical corticosteroids, antihistamines, pest control	No
Cutaneous larva migrans	1	52	M	Albendazole 400 mg daily × 7 days	No
Subcutaneous dirofilariasis	1	72	F	Surgical excision + albendazole 800 mg daily × 28 days	No
*Dermanyssus gallinae* dermatitis	1	70	M	Topical treatment, pest control, management of farm and companion animals	No
Pediculosis	32	9	28 F/4 M	Mechanical removal; permethrin, pyrethrins, lindane; resistant case treated with topical permethrin + oral ivermectin	Yes (n = 1, 3.1%)
Tick bites (incl. Lyme disease)	25	46	18 M/7 F	Systemic antibiotics in Lyme cases (n = 7, 28% of tick bites)	No
Myiasis	1	41	F	Larval extraction; disinfection; systemic antibiotic prophylaxis	No
Scabies	3	24	3 M	Benzyl benzoate failure → topical permethrin + oral ivermectin	Yes (100% of scabies cases, n = 3)
Cutaneous leishmaniasis	1	30	M	Antimonial therapy (weekly × 4 weeks)	No
Eosinophilic dermatosis of haematologic malignancy (EDHM)	1	51	M	Supportive/symptomatic management	N/A

**Table 2:** Literature-based overview on some of the most important ectoparasite and vector-borne-related dermatoses, with a focus on Europe.

Skin Condition (Aetiological Agent)	Epidemiology & Risk Factors	Clinical/Diagnostic Features	Suspected Resistance/Failure Reports	Standard Therapy
Flea bites (order Siphonaptera, different genera involved).	Common worldwide, esp. warm/humid areas; zoonotic, frequent with domestic animals/rodents/livestock [15,16,17,18].	Grouped erythematous papules with central punctum; dermoscopy: annular ecchymotic macules. Typical distribution on lower limbs or other exposed areas. In sensitised individuals, lesions may evolve into papular urticaria or flea allergy dermatitis; scratching may lead to secondary bacterial infection [15,16,17,18].	Documented resistance against pyrethroids in humans and animals [19].	Symptomatic: corticosteroids, antihistamines; treat animals + environment disinfestation. Topical or systemic antibiotics may be required if superinfection occurs [17,18].
Bed bug dermatitis (*Cimex lectularius* and *C. hemipterus*).	Resurgence since 1990s; linked to travel + insecticide resistance. Infestations affect hotels, dormitories and hospitals, regardless of hygiene [20,21,22].	Linear papules (breakfast–lunch–dinner sign); clinical diagnosis + environmental inspection. Reactions may be associated with anaemia, sleep disturbances and psychological distress [21,22].	Increasing pyrethroid resistance worldwide [20,21,22].	Symptomatic therapy + integrated pest control. Integrated strategies include laundering, vacuuming, extreme temperatures and insecticides [21,22].
Cutaneous larva migrans (CLM; *Ancylostoma braziliense*).	Frequent in tropics; imported in Europe. Most commonly caused by *Ancylostoma braziliense*; transmitted through contact with contaminated soil or sand [23,24].	Serpiginous, pruritic erythematous tracks; dermoscopy: whitish tracks. Lesions usually self-limited but intensely pruritic [24].	No resistance reported.	Albendazole, ivermectin; mebendazole [24,25].
Dirofilariasis (*Dirofilaria repens*, *D. immitis*).	Endemic in Europe; zoonotic, climate-related. Human infection typically caused by *Dirofilaria repens*, rarely *D. immitis*. Transmission via mosquito bites [26,27,28,29,30].	Subcutaneous nodules; histology confirms. Occasional involvement of ocular, pulmonary or CNS sites has been reported [28,29,30,31,32].	Resistance to macrocyclic lactones has been described in dogs [33,34].	Surgical excision; albendazole in selected cases. Antiparasitic therapy may be considered in cases of incomplete excision or suspected persistence [28,29,30].
*Dermanyssus gallinae* dermatitis.	Avian mite also known as the red poultry mite; zoonotic, linked to pigeons/bird nests and poultry; underdiagnosed. Infestations may spread indoors when birds are absent, or in housing close to backyard poultry farms [35,36,37,38,39].	Pruritic maculo-papular/vesicular lesions, often misdiagnosed as scabies. Lesions often involve trunk and nuchal region; mites can be identified microscopically [36,37].	Not relevant (no systemic drugs).	Source eradication with removal of bird nests from the building to prevent mite migration indoors; cleaning, targeted acaricide treatment applied to cracks, crevices of the walls; hot water washing of bedding; antihistamines, corticosteroids [37,38].
Human pediculosis (*Pediculus humanus capitis, Pediculus humanus corporis, Pthirus pubis*).	Head lice (*Pediculus humanus capitis*) are common in children; body lice (*Pediculus humanus corporis)* are more frequent in people living in crowded conditions or with poor hygiene; pubic lice (*Pthirus pubis*) are sexually transmitted. Body lice are known vectors of epidemic typhus and trench fever [40,41].	Scalp pruritus, excoriations, lymphadenopathy; dermoscopy aids species ID. Pubic lice infestation of eyelashes may be diagnosed dermoscopically [40,42].	Global pyrethroid resistance ~60%, up to 82% post-2015 [43,44].	Combing; permethrin, pyrethrins, malathion; ivermectin/dimethicone in resistant cases. Occlusive agents such as petroleum jelly may be used for eyelashes [42,45].
Lyme disease (*Borrelia burgdorferi*).	Endemic in Europe, N. America, Asia. *Ixodes* spp. ticks transmit *Borrelia burgdorferi* sensu lato complex. Zoonotic [46,47].	Erythema migrans hallmark; clinical + serology. Disease may progress to neurologic, cardiac and arthritic involvement [47].	Antibiotic-refractory arthritis possible [48].	Doxycycline or Amoxicillin; ceftriaxone for severe neuro/cardiac cases. Prolonged antibiotics not recommended in refractory arthritis; immunomodulatory strategies may be considered [47,48].
Myiasis (main agents: *Dermatobia hominis, Cordylobia anthropophaga, Lucilia* spp., *Wohlfahrtia magnifica*).	Associated with international travel; autochthonous cases are rare in Europe, except for *Wohlfahrtia,* which causes common myiasis in animals [49]. Caused by larvae of various Diptera species (*Dermatobia hominis, Cordylobia, Lucilia, Wohlfahrtia*). Zoonotic [50,51,52,53,54].	Furuncular nodules with central pore; dermoscopy shows larvae. Other forms include migratory or wound-associated lesions [52,53,54].	No resistance data.	Larval removal (occlusion/extraction); ivermectin if extensive. Manual extraction should avoid larval rupture to prevent granuloma formation [53,54].
Scabies (*Sarcoptes scabiei* var. *hominis*).	Affects > 130 M globally; WHO neglected tropical disease. Transmission occurs primarily through close skin-to-skin contact; fomite transmission possible. Rarely zoonotic [55,56,57].	Pruritus, burrows, papules; dermoscopy shows mites/burrows. Secondary bacterial infection is common, particularly in children, immunocompromised and elderly patients [56,58].	Treatment failure 15.2% overall; benzyl benzoate 25.3%, crotamiton 27.7%; rising permethrin/ivermectin failures. Resistance remains difficult to quantify due to lack of standardised susceptibility testing [59].	Permethrin, ivermectin; alternatives: benzyl benzoate, crotamiton; combos in resistant cases. Two doses of ivermectin reduce failure rate compared to a single dose [59,60].
Cutaneous leishmaniasis (*Leishmania infantum*).	600 k–1 M new cases/year; endemic in dogs in Southern Europe. Transmission via female sandflies (*Phlebotomus* in Old World, *Lutzomyia* in New World); zoonotic [61,62,63,64,65,66,67].	Papules, nodules, ulcers; dermoscopy: yellow tears, vascular structures. Lesions may be ulcerative, verrucous, eczematous or nodular depending on species and host response [62,64].	Antimonial resistance rising in India/Middle East [68].	Antimonials, amphotericin B, miltefosine, cryotherapy. Cryotherapy is an alternative for selected cases [61,62,63,68].

## Data Availability

The aggregated data supporting the findings of this study are included within the article. De-identified individual-level data underlying the case series are available from the corresponding author upon reasonable request. The data are not publicly available due to privacy and ethical considerations.

## Supplementary Materials

The following supporting information can be downloaded at: https://www.mdpi.com/article/10.3390/jcm15020851/s1, Table S1: Descriptive statistics and prevalence of parasitic and arthropod-related dermatoses (n = 88), Figure S1: Diagnostic approach for suspected ectoparasite or vector-borne-related dermatoses.
