# Supplementary material for: Ectoparasite- and Vector-Borne-Related Dermatoses: A Single-Centre Study with Practical Diagnostic and Management Insights in a One Health Perspective
Source: J Clin Med. 2026 Jan 20;15(2):851. doi: 10.3390/jcm15020851 (PMC12841827; doi:10.3390/jcm15020851)
Supplement: Supplementary file 1 [file jcm-15-00851-s001.zip › jcm-4045912-supplementary.pdf]

SUPPLEMENTARY MATERIAL

Table S1. Descriptive statistics and prevalence of parasitic and arthropod-related dermatoses (n = 88)

Condition	n (%)	Mean age (years)	Male (%)	Female (%)	Observed resistance
Pediculosis	32 (36.4%)	9	12.5	87.5	1 (3.1%)
Tick bites (incl. Lyme disease)	25 (28.4%)	46	72.0	28.0	0
Bed bug bites	18 (20.5%)	32	44.4	55.6	0
Flea bites	4 (4.5%)	32	75.0	25.0	0
Scabies	3 (3.4%)	24	100.0	0	3 (100%)
Cutaneous larva migrans	1 (1.1%)	52	100.0	0	0
Subcutaneous dirofilariasis	1 (1.1%)	72	0	100.0	0
Dermanyssus gallinae dermatitis	1 (1.1%)	70	100.0	0	0
Myiasis	1 (1.1%)	41	0	100.0	0
Cutaneous leishmaniasis	1 (1.1%)	30	100.0	0	0
EDHM	1 (1.1%)	51	100.0	0	N/A
Total	88 (100%)	28.8 (mean)	45.5	54.5	4 (4.5%) overall

Among the 88 patients included, the most frequent conditions were pediculosis (36.4%), tick bites (28.4%), and bed bug bites (20.5%), followed by less common parasitic dermatoses. The overall mean age was 28.8 years (range 1–72), with a slight female predominance (54.5%). Resistance to standard therapy was documented in 4 patients (4.5% overall), corresponding to one pediculosis case (3.1% of lice infestations) and all three scabies cases (100% of scabies infestations).

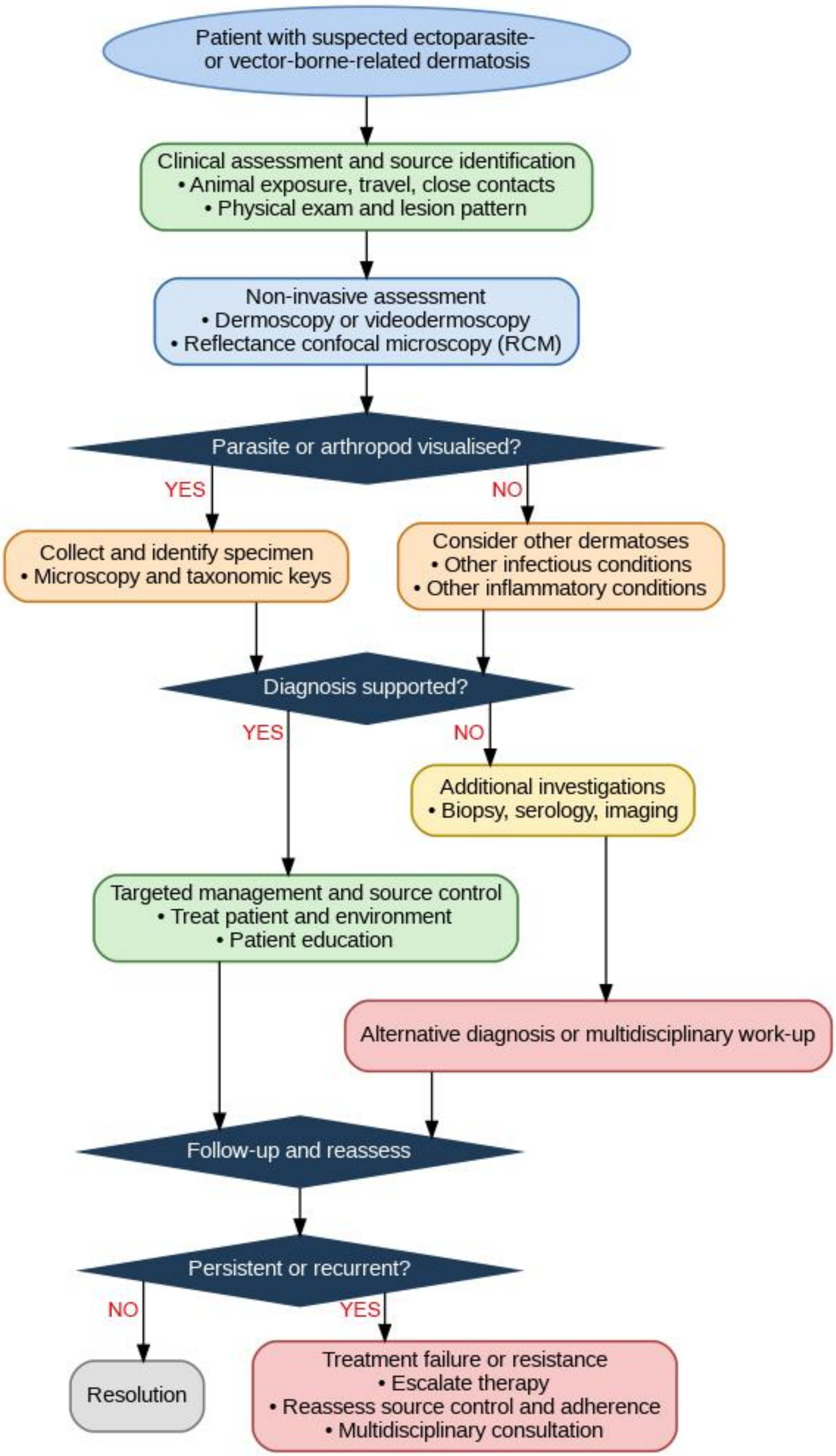

Figure S1. Diagnostic approach for suspected ectoparasite or vector-borne-related dermatoses.
